# The Roles of Cardiac Fibroblasts and Endothelial Cells in Myocarditis

**DOI:** 10.3389/fcvm.2022.882027

**Published:** 2022-04-07

**Authors:** Yunling Xuan, Chen Chen, Zheng Wen, Dao Wen Wang

**Affiliations:** ^1^Division of Cardiology, Department of Internal Medicine, Tongji Hospital, Tongji Medical College, Huazhong University of Science and Technology, Wuhan, China; ^2^Hubei Key Laboratory of Genetics and Molecular Mechanisms of Cardiological Disorders, Wuhan, China

**Keywords:** myocarditis, cardiac fibroblasts, endothelial cells, cross-talk, extracellular vesicles

## Abstract

In myocarditis caused by various etiologies, activated immune cells and the immune regulatory factors released by them play important roles. But in this complex microenvironment, non-immune cells and non-cardiomyocytes in the heart, such as cardiomyocytes (CMs), cardiac fibroblasts (CFs) and endothelial cells (ECs), play the role of “sentinel”, amplify inflammation, and interact with the cardiomyocytes. The complex interactions between them are rarely paid attention to. This review will re-examine the functions of CFs and ECs in the pathological conditions of myocarditis and their direct and indirect interactions with CMs, in order to have a more comprehensive understanding of the pathogenesis of myocarditis and better guide the drug development and clinical treatment of myocarditis.

## Introduction

The pathological standard of myocarditis refers to the infiltration of inflammatory cells, with or without myocardial necrosis in the staining of heart tissue sections according to the Dallas definition (1). A recent study estimates that the global prevalence of myocarditis is ~22 out of 100,000 patients per year (2). A variety of infectious pathogens, systemic diseases, drugs, and toxins can cause the disease (3). Myocarditis can appear with a variety of different characteristics, from mild symptoms of chest pain to ventricular arrhythmia or fatal cardiogenic shock (3). Inflammatory cells and inflammatory factors in innate immunity and adaptive immunity play important roles in the occurrence and development of myocarditis, and there has been review (4) clarifying the roles of various types of immune cells. However, the vast majority of the heart is non-inflammatory cell. As our understanding of the human immune response continues to increase, many cell types, such as CFs, ECs that were initially thought to be just bystanders have been proven to play vital roles in the inflammatory process and even determine the direction and intensity of the immune response. However, there is little research on the role of non-inflammatory cells in the process of myocarditis. Thus, in this review, we will briefly delineate the role of non-inflammatory cells, non-CMs including CFs and ECs, and focus on the everlasting cross-talk between CFs, ECs and CMs in the progression of cardiovascular diseases.

## CFs

**Figure 1:**
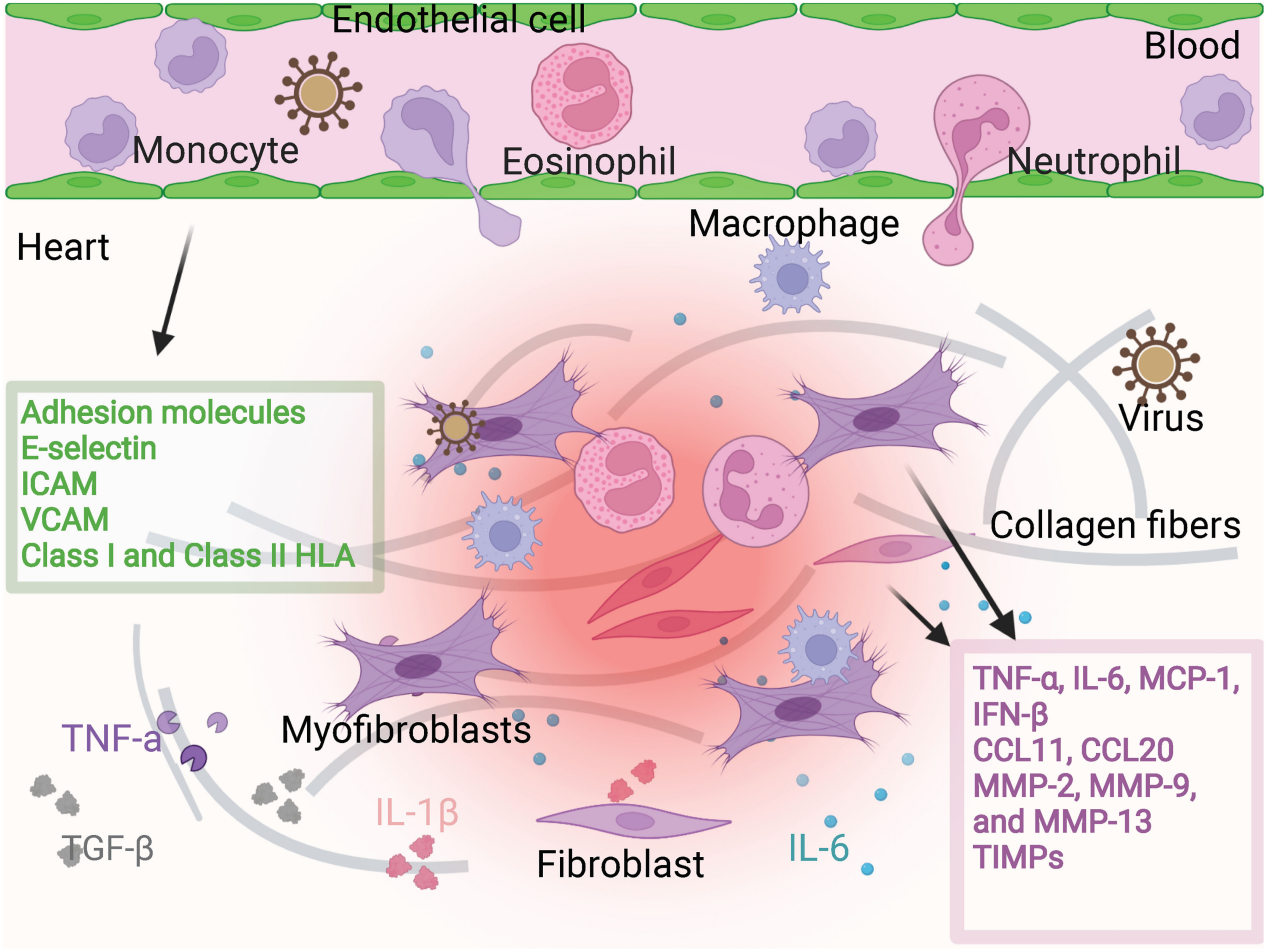
Fibroblast and endothelial cell activation. Fibroblasts are activated in viral infection or in the environment containing cytokines. Then they produce pro-inflammatory factors and chemokines such as TNF-α, IL-6, MCP-1, IFN-β, CCL11, CCL12. They can also produce MMP family proteins to regulate cardiac interstitial fibrosis. Under the stimulation of these microenvironments, endothelial cells produce adhesion molecules to regulate the adhesion and infiltration of inflammatory cells. The participation of fibroblasts and endothelial cells promotes more severe cardiac inflammatory infiltration and more severe fibrosis.

Fibroblasts in the adult mouse heart make up about 15% of the non-myocytes in the heart (5). The basic function of cardiac CFs is to synthesize a collagen-rich ECM network to provide structural integrity and biomechanical signals (6). They are dynamic participants in ventricular physiology and pathophysiology. CFs can be activated either by their infection or invasion (7, 8) or by heart stress. Activated CFs will secret inflammatory factors, chemokines, or other immunomodulatory substances and produce large amounts of ECM. In myocarditis, virus infection or cytokines induces fibroblast activation state and then CFs function through virus transmission, cell damage, chemokines, and inflammatory factors production, making heart inflammation to keep getting worse. At the same time, the extracellular matrix produced by CFs promotes myocarditis from acute inflammatory infiltration to the chronic phase (Figure 1).

### Pathogenic Infection of CFs

CFs can directly serve as the host of the virus. Lindner et al. demonstrated that when both CMs and CFs are infected with coxsackie virus B3 (CVB3), the viral replication within CFs increases by 10-folds, indicating that they play a key role in promoting viral load in myocarditis (9). Within 9 h after infection, the CMs network loses its ability to contract spontaneously, and then decomposes and is replaced by overgrown CFs that survive from the infection (10).

Infected CFs can continue to damage the heart function at different stages of myocarditis. In the acute phase of viral myocarditis, after CFs are attacked and die, the virus in the cell continues to attack various types of cells, thereby making it a storage and transmission reservoir for the virus in the infection (11). While in the chronic phase, the virus that resides in CFs may be an important cause of persistent inflammation, heart dilation, and even heart failure. In addition, viral infection can cause damage to CFs and their release of damage-associated molecular patterns (DAMPs), such as HGMB1 (12).

### Inflammation Reaction Caused by CFs

Although CFs are not a component of the immune system, they play an important role in the occurrence and development of inflammation. The expression of chemokines has been found in a variety of disease processes related to tissue damage and leukocyte recruitment.

Eosinophils are multifunctional granulocytes that help trigger and regulate inflammation. It is reported that eosinophils aggravate the pathological severity and mortality of eosinophilic myocarditis (13). In general, the transport of eosinophils to the heart in eosinophilic myocarditis depends on the expression of the chemokine receptor CCR3 (14) and its ligands CCL11, CCL24, and CCL26 in the mouse model and the heart of patients with eosinophilic myocarditis. However, CFs produce CCL11 under the regulation of two cytokines IL-4 and IL-13 ADDIN EN.CITE during myocarditis (15).

IL-17 is involved in the pathogenesis of autoimmune myocarditis, and it has been shown that neutralizing IL-17 can reduce the severity of myocarditis (16). *In vitro*, the stimulation with TNF-α, IL-1β, and IL-17 can induce CFs to produce CCL20. CCL20 promotes adhesion of Th17 on endothelium and induces Th17 cell migration (17), thereby producing more IL-17 to form a vicious circle. What's more, IL-17A acts on the differentiation of Ly6C (low) monocytes into macrophages through CFs-derived GM-CSF *in vitro*, indicating that CFs promote monocyte differentiation and proliferation, and regulate monocytes and monocyte-derived macrophages phenotype and function (18).

The continuous expression of cytokines was observed in the mouse model of dilated cardiomyopathy (DCM) after myocarditis. Compared with other cytokines, the expression of the IL-1β gene in the chronic phase was relatively higher, and it was related to the ratio of heart weight/body weight and the degree of fibrotic lesions (19). IL-1β secreted by CFs initiates the inflammatory process and attracts proinflammatory immune cells. In addition, CFs-secreted IL-1β leads to the maintenance of inflammation in the later stage of wound healing (20).

Overall, in myocarditis caused by a variety of factors, CFs can participate in the entire inflammatory reaction process and even the chronic phase by secreting chemokines or inflammatory factors. Most of the inflammation involved in CFs is harmful, but there are also beneficial parts, so it is particularly important to find the reason for these differential effects and carry out corresponding interventions.

### The Change of ECM Leads to the Formation of Fibrosis

The ECM components in the interstitium of the heart form a complex network structure, which provides structural support for several different cell types, and integrates extracellular signals and cellular responses (21). The ECM is a highly dynamic structure that exists in all tissues and undergoes continuous controlled remodeling. In the body, CFs are considered to be a key regulator of ECM structure. CFs residing in the heart are not only the main producer of ECM components but also the main source of matrix metalloproteinases (MMPs) and tissue inhibitors of MMPs (TIMPs). In inflammatory heart disease, cytokines, growth factors can change ECM components and the production of MMPs and TIMPs by stimulating the migration and proliferation of CFs or mediating the interaction between CFs and other cell types (22).

For example, TNFα and IL-1β lead to increased expression of MMP-2, MMP-9, and MMP-13 in CFs (23) which cause ECM remodeling. On the one hand, prolonged activation time of CFs and progressive fibrosis can lead to further deformation of the tissue structure and deterioration of heart function. ECM remodeling during myocarditis can cause arrhythmia and even heart failure. On the other hand, ECM components may adsorb parasite antigens (24) and cytokines (25) that may contribute to the establishment and continuation of inflammation.

The regulation of matrix metalloproteinases (MMPs) in the acute and chronic phases of viral myocarditis and its role in myocardial interstitial remodeling have been reviewed (26) in detail. Therefore, adjusting the function of CFs to regulate cardiac inflammation and cardiac remodeling is gradually turning into a better solution.

## ECs

ECs are arranged in the circulatory system and are essential for maintaining and regulating vascular homeostasis (27) by protecting material transport, controlling vascular permeability, and regulating vascular tension (28). They are the largest cell population in non-cardiomyocytes of the adult mouse heart, accounting for about 60% (5). Resting ECs isolate leukocyte-interacting proteins in specialized secretory vesicles (29), so they cannot interact with white blood cells. In addition, resting ECs also inhibit the transcription of other adhesion molecules, such as E-selectin, vascular cell adhesion molecule 1 (VCAM1), and to a large extent inhibit the transcription of intercellular adhesion molecule 1 (ICAM1) and pro-inflammatory cytokines (30).

However, under the influence of certain factors, ECs can be transformed into an activated state. In DCM developed from autoimmune myocardial inflammation, it is shown that endothelial activation in organ-specific diseases affects the function of blood vessels throughout the body. Vallbracht et al. proved this and found that patients with myocardial inflammation showed impaired endothelial function of radial artery, and these patients have ruled out the risk factors of classic arterial damage, such as coronary artery disease, severe left ventricular failure, diabetes or atherosclerosis (31). For myocarditis, myocardial inflammatory infiltration, endothelial activation, direct viral toxicity, and circulating cytokines cause endothelial dysfunction in patients with persistent myocardial viruses (32). The persistence of the myocardial virus can independently cause endothelial dysfunction. For patients with myocardial leukocyte infiltration, endothelial dysfunction is more obvious (32). Nearly half of patients with DCM have increased T lymphocyte density and increased immune activation of ECs and mesenchymal cells in cardiac biopsies (33).

ECs not only act as a transport device for immune cells in circulation, forming a mechanical barrier to resist invaders, but also produce chemokines, interleukins, interferons, and growth factors through paracrine after they are activated. In addition, they can induce the expression of adhesion molecules such as E-selectin, P-selectin, ICAM, or VCAM to attract and transfer immune cells to inflammatory sites (34) (Figure 1).

### Pathogenic Infection of ECs

Many pathogenic microorganisms not only infect CMs and CFs, but also the ECs. Among *T. cruzi* and more than 20 viruses related to myocarditis, they are known to infect human and/or animal ECs (35). Parvovirus B19 is considered the pathogen in most cases of chronic myocarditis (36). And PVB19-related inflammatory cardiomyopathy is characterized by infection of the ECs of the heart in small arteries and veins (37).

Bültmann et al. reported (38) a patient with severe myocarditis, whose radioactive *in situ* hybridization detected the viral genome in ECs, but did not detect the viral genome in CMs or other tissue components. What's more, humans or mice infected with PVB19 show more particles related to endothelial cell damage in the blood circulation (39). Due to the high viral load in cardiac ECs, PVB19 infection of ECs is sufficient to induce coronary microcirculation damage and secondary CMs necrosis (40).

However, ECs, like a barrier that the virus needs to cross, can to a certain extent provide a buffer for the time when the heart damage occurs so that the immune system can have a longer time to exert a stronger immune effect before the virus invades the CMs (41).

### Inflammation Reaction Caused by ECs

Adhesion molecules (42) are a class of membrane receptors with various functions, including cell migration, cell-cell interaction, and cell-to-extracellular matrix adhesion. They are involved in various processes such as growth, differentiation, migration, and apoptosis. The adhesion molecules VCAM-1, ICAM-1, and E-selectin expressed on the vascular endothelium act as ligands for the counter-receptors of circulating inflammatory cells (43).

The expression of endothelial cell surface markers is dynamic and is affected by circulating cytokines and other stimuli (44). The inflammation state of endomyocardial biopsy in patients with dilated cardiomyopathy was evaluated by immunohistochemistry. It was found that 45.8% of patients had endothelial inflammation activation, which was manifested by increased expression of at least three adhesion molecules (Class I and Class II HLA, ICAM-1, VCAM-1) (45).

The increased expression of endothelial adhesion molecules in the myocardium of patients with infectious myocarditis may be related to pro-inflammatory cytokines. TNFα and IL-1β increase the expression of adhesion molecules ICAM-1, VCAM-1 and E-selectin on the ECs of the human coronary artery and cardiac microvascular system (46). Cytokines and chemokines driven by *T. cruzi* can also regulate adhesion molecules like VCAM-1 and ICAM-1 on the ECs of target tissues, and play a key role in cell recruitment (24).

## Cross-Talk Among CFs, ECs, and CMs: Direct and Indirect Interaction

**Figure 2:**
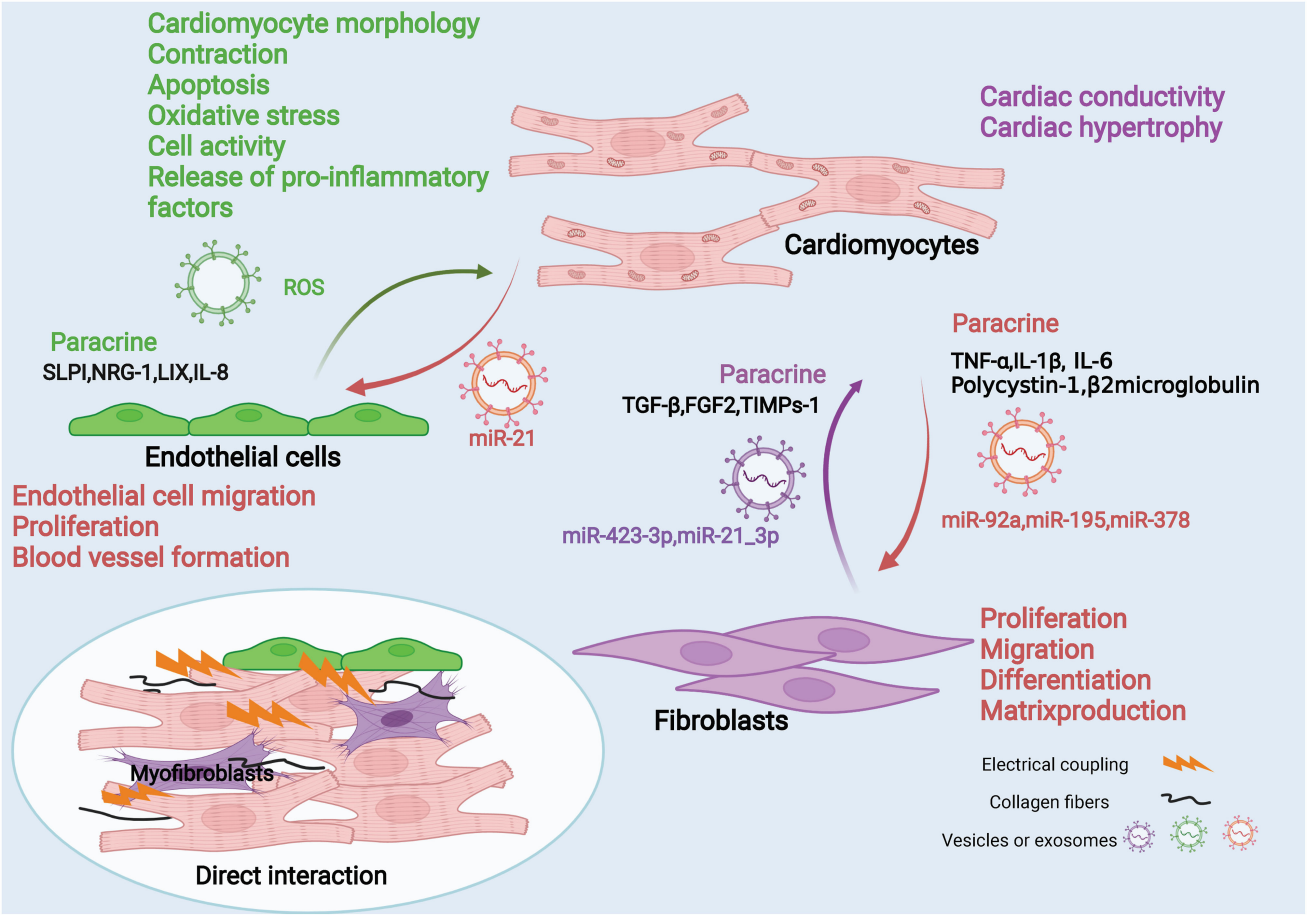
Fibroblasts, endothelial cells and cardiomyocytes interaction diagram. Fibroblasts, endothelial cells can interact with cardiomyocytes through mechanical coupling and electrical coupling. They also act through paracrine effects and/or cell-secreted vesicles or exosomes. Cardiomyocytes secrete pro-inflammatory factors such as TNF-α, IL-1β, and IL-6 through paracrine effects to act on fibroblasts, and they can also deliver intracellular miRNAs through exosomes, such as miR92a, miR195, and miR378. These mediators can affect fibroblast proliferation, migration, differentiation and matrix production. Fibroblasts can also secrete factors such as TGF-β through paracrine action or deliver miRNAs into cardiomyocytes through exosomes, which affect cardiomyocyte contraction and cardiac hypertrophy. Similarly, endothelial cells can secrete effector molecules such as IL-8 through paracrine effects, and can also carry ROS through exosomes, thereby affecting various functions of cardiomyocytes. In contrast, cardiomyocytes can affect endothelial cell migration, proliferation, and angiogenesis by secreting exosomes carrying molecules such as miR-21.

The functions of various cells are not independent, they share the characteristics of repetitiveness and mutual reinforcement or containment. CMs are the most important cell type for the heart to perform biological functions. So we focus on the interaction between CMs and CFs, and CMs and ECs (Figure 2). On the one hand, these interactions promote the heart's adaptive response to internal and external stimuli and compensate or overcome these pressures. On the other hand, these interactions can also cause pathological remodeling related to heart disease, speed up the disease process, and lead to heart failure and cardiogenic death. Therefore, the study of the interaction between cells will help us to employ a synergistic protective effect between cells and avoid the expansion of malignant events.

### Direct Cell-to-Cell Interaction

There are mechanical coupling and electrical coupling (47) between CMs, CFs, and ECs. This kind of spatial connection enables the direct exchange of information between each other. Research shows blocking contact between CMs and CFs using antibodies against cardiac fibroblast plasma membrane protein or connexin will inhibit cardiomyocyte adhesion and reduce IL-6 production, but will not reduce TNFα production (48). However, the direct interaction is very dependent on the tight connection in space. Compared with the indirect action, its range of action is smaller. Therefore, we mainly discuss the indirect interaction between cells.

### Indirect Cell-to-Cell Interaction: Paracrine Effects of Released Cytokines/Chemokines or Others

#### CF-CM and CM-CF

The interaction between CFs and CMs leads to paracrine of regulatory factors, which in turn affects cardiac conductivity, and hypertrophy, contraction (49), and apoptosis of CMs. While CMs change the proliferation, migration, differentiation, and matrix production capacity of CFs through paracrine (Table 1).

**Table 1:** Table of CF-CM and CM-CF interactions in the cardiovascular field.

**Disease or treatment**	**Cross-talk**	**Mode of action**	**Acting molecule**	**Cell effects**	**References**
Heart development	CF-CM	Paracrine	–	CMs are specialized as ventricular conduction system-like cells, CNMs maturation	(50, 51)
Heart failure caused by TAC	CM-CF	Paracrine	IL-6	CFs migration, proliferation, and myofibroblast differentiation	(52)
	CM-CF	Extracellular vesicles	miR-378	CFs proliferation, fibrosis	(53)
	CM-CF	Paracrine	β2 microglobulin	CFs activation	(54)
MI	CM-CF	Extracellular vesicle	miR-92a	Myofibroblast activation	(55)
–	CM-CF	Exosomes	miR-195	Myofibroblast activation	(56)
	CF-CM	Paracrine	TGF-β	CMs hypertrophy	(57)
DOX-induced cardiotoxicity	CF-CM	Paracrine	FGF2	CMs damage	(58)
Early hypoxia	CM-CF	Paracrine	TNF-α, IL-1β	CFs migration	(59)
Ang II-induced cardiac hypertrophy	CF-CM	Exosomes	miR-21-3p	CMs hypertrophy	(60)
H/R	CF-CM	Paracrine	TIMPs-1	CMs viability	(61)
	CF-CM	Exosomes	miR-423-3p	CMs viability and apoptosis	(62, 63)
	CM-CF	Paracrine	Polycystin-1	CFs differentiate, profibrotic factors expression	(54)

–, *No specific information is given in the original text*.

*TAC, transverse aortic constriction; MI, myocardial infarction; DOX, Doxorubicin; FGF2, fibroblast growth factor 2; H/R, Hypoxia–reoxygenation; Ang II, angiotensin II; Gal-3, Galectin-3; sFRP2, Secreted frizzled-related protein 2*.

For example, TGF-β1 is the prototype of a large family of structurally related secreted dimeric proteins. It is pleiotropic and plays an important role in intercellular signal transduction (64). Disturbance of TGF-β action can lead to pathological conditions, including cardiovascular disease (65), fibrotic disease, and cancer (66). When subjected to mechanical stretching, TGF-β secreted by CMs and CFs induces the growth response of CMs and CFs in an autocrine/paracrine manner. In addition, the inflammatory factors released by immune cells will also promote the release of TGF-β from CFs. The increase of TGF-β will promote the protein synthesis rate of static CMs (67) and change the function of CMs.

In cardiac remodeling, myofibroblasts can induce CMs hypertrophy through cross-talk between CFs and CMs (68). IL-6 signaling in CMs co-cultured with CFs is activated to promote CMs hypertrophy (69). In addition, when CMs are co-cultured with cardiac CFs, the production of tumor necrosis factor (TNF)-α is also upregulated (48).

Similarly, CMs also have a paracrine effect on CFs. For example, the pro-inflammatory cytokine hypoxia-induced mitotic factor (HIMF) was overexpressed in CMs *in vitro*, and the cell supernatant was used as a conditioned medium and co-cultured with CFs. The results (52) show that CMs induce CFs migration, proliferation, and myofibroblast differentiation by paracrine IL-6.

#### EC-CM and CM-EC

Since ECs and CMs are relatively close in space, they can conduct direct cell signal transduction and two-way communication through paracrine. The interaction between CMs and ECs is essential for heart development, postnatal function, and heart repair (70). ECs influence cardiomyocyte morphology, contractility, apoptosis, oxidative stress, cell activity, and release of pro-inflammatory factors in a paracrine manner. At the same time, CMs can promote endothelial cell migration, proliferation, and blood vessel formation in the same way (Table 2).

**Table 2:** Table of EC-CM and CM-EC interactions in the cardiovascular field.

**Disease or treatment**	**Cross-talk**	**Mode of action**	**Acting molecule**	**Cell effects**	**References**
ET-1 pre-stimulated ECs	EC-CM	Paracrine	–	CMs hypertrophy	(71)
Hypoxic ECs	EC-CM	Paracrine	TGFβ1	CMs apoptosis	(72)
H/R	EC-CM	Paracrine	SLPI	CMs damage	(73)
	EC-CM	Microvesicles (MVs)	ROS	CMs apoptosis and oxidative stress	(74)
OGD	CM-EC	Exosomes	miR-21	Angiogenic activity	(75)
Ischemic preconditioning	EC-CM	Exosomes	–	CMs activity	(76)
–	hiPSC-CM -EC	Exosomes	miR-21-3p	Angiogenesis, ECs migration and proliferation	(77)
LPS	EC-CM	Exosomes	–	CMs damage	(78)
DCM	EC-CM	Paracrine	NRG-1	CMs contractility	(79)
Hypercholesterolemia	EC-CM	Paracrine	Lipopolysaccharide-induced chemokine (LIX) and IL-8	CMs apoptosis, Expression of the proinflammatory cytokines	(80)

–*, No specific information is given in the original text*.

*ET-1, endothelin-1; SLPI, Secretory leukocyte protease inhibitor; ROS, Reactive Oxygen Species; OGD, Oxygen–glucose-deprivation; hiPSC-CM, Human induced pluripotent stem cell-derived cardiomyocytes; LPS, Lipopolysaccharide; NRG-1, Neuregulin-1*.

For example, Endothelin-1 (ET-1) is described as a 21 amino acid peptide produced by ECs and is the most effective known endogenous vasoconstrictor. CMs mainly express ETA receptors. Under normal physiological conditions, ET-1 promotes the contraction of CMs. However, under pathological conditions, increased ET-1 can damage the contractile function of CMs through ETA receptors (81).

In addition to the direct effect of substances secreted by one type of cell on another type of cell, the substances secreted by cells can affect them through synergistic or antagonistic effects with other cells. Pummerer et al. showed that normal hearts are not easily affected by T cells that react with myosin (82). In myocarditis, the cardiac mesenchymal cells expressing MHC class II are significantly increased and the expression of endothelial ICAM-1 is strongly upregulated, making the heart vulnerable to passive metastasis and myosin responds to T cell attack.

In general, ECs and CMs can communicate extensively through secreted substances or “permissible action”, which is essential for the overall functioning of the heart.

### Indirect Cell-to-Cell Interaction: Extracellular Vesicles-A New Pathway of Communication

The above-mentioned paracrine substances can play a corresponding role through the binding of receptors on the corresponding target cells. These substances are of fewer types and have a limited range of action. However, the production of EVs, as a new pathway of communication, enriches the medium of communication and expands the distance of interaction between cells.

EVs are cell-derived membrane structures that are secreted or shed from the plasma membrane after fusion of the endosome with the plasma membrane. Exosomes (83), as the current hottest research type in EVs, are membrane vesicles that carry biologically active molecules such as proteins, lipids, messenger RNA (mRNA), and micro RNA (miRNA) which mediate intercellular communication between different cell types within the body, thereby affecting normal and pathological states. And they have become a key medium for intercellular communication. Exosomes can be induced by many cell events including cell death, hypoxia, stress, oncogene expression, differentiation, and viral infection (84). They deliver downstream signals in the following three ways: cell internalization, direct fusion with the cell membrane, and receptor-ligand interaction (85). All major heart cell types including CMs, ECs, and CFs release exosomes to regulate cell function. What is special is that the protein content of cardiac exosomes is significantly different from other types of exosomes in the literature, which contain cytosolic, sarcomeric, and mitochondrial proteins (86).

For example, CFs-derived exosomes mediate crosstalk between CFs and CMs and lead to cardiomyocyte hypertrophy (60) via internal miRNA. Exosomes isolated from hypoxic CMs promote CFs apoptosis and inhibit their proliferation, migration, and invasion (87) via internal lncRNA.

In addition, exosomes can transfer genetic material between cells. CVB can cause a variety of life-threatening inflammatory diseases. It is a member of the picornaviridae family and is a non-enveloped single-stranded RNA virus related to human diseases including myocarditis (88) and pancreatitis. Although it is well-known that CVB spreads through cytolysis, recent reports reveal a second route in which CVB can be encapsulated in host membrane components and escape the cell in the form of exosome-like particles (89). Recent evidence suggests that CVB and other picornaviruses hijack the host membrane and obtain the envelope. Obtaining the envelope may provide unique benefits for CVB virus particles, such as resistance to neutralizing antibodies and effective non-lytic virus transmission (90). While EVs participate in the transmission of HSV-1, in which microvesicles (MVs) containing virus particles released by infected cells are endocytosed by immature cells, leading to productive infection (91).

## Targeting Fibroblasts or Endothelial Cells in the Treatment of Myocarditis

As mentioned above, fibroblasts are activated under the influence of viruses or cytokines. Therefore, reducing the invasion of viruses in fibroblasts and reducing the stimulation of fibroblasts by other inflammatory factors are important means to prevent their activation. The study by Heim et al. showed that Ribavirin, the antiviral drug, has high cell-specific activity in fibroblasts, and it can reduce the viral load in fibroblasts (92). Moreover, Kania et al. demonstrated TGF-β-mediated myofibroblast differentiation and progression of myocardial fibrosis in human and mouse myocarditis (93). Anti-TGF-β treatment prevented myocardial fibrosis in mice with autoimmune myocarditis. In addition, IL-1 can prompt uninfected fibroblasts to enhance the induction of inflammation-related genes in the presence of infected fibroblasts. Blockade of IL-1 receptor signaling with an IL-1 receptor antagonist and knockdown of IL-1 receptor using siRNA abolished cytokines in human fibroblasts during CVB3 infection (94).

Fibroblasts can produce a variety of inflammatory factors and chemokines after activation. Among them, CCL20 is a chemokine that can effectively recruit Th17 cells. The neutralization of CCL20 can effectively reduce cardiac inflammation (17). In addition, blocking HMGB1 secreted by fibroblasts can effectively improve cardiac fibrosis in EAM mice (12). Studies have shown that fibroblasts in the heart are a heterogeneous cell population, in which Sca-1+ cardiac fibroblasts are not only efficient GM-CSF producers, but also have certain plasticity (95). They have a certain degree of plasticity to alter their own cytokine production under the influence of local microenvironment. Therefore, exploring the characteristics of different subpopulations of fibroblasts in myocarditis and finding targeted interventions based on their characteristics can better inform the treatment of patients with myocarditis.

After endothelial cells are infected by virus, the expressions of cell adhesion molecules such as ICAM-1, integrin αvβ3, P- and E-selectin are increased and the release of coagulation factor-von Willebrand factor (VWF) is also increased (96). The increase of adhesion molecules makes the circulating inflammatory cells more likely to adhere to blood vessels, while the increase of coagulation factors aggravates the retention of inflammatory cells and affects the execution of vascular function through the coagulation pathway. Telbivudine, an antiviral drug clinically used to treat hepatitis B, exerts endothelial protection in HPV B19 infected endothelial cells and can improve HPV B19-associated inflammatory cardiomyopathy (97). In addition, CVB3 virus can also infect endothelial cells through Coxsackie and adenovirus receptor (CAR). Bosentan and valsartan are angiotensin II receptor antagonists for the treatment of hypertension. In CVB3-induced myocarditis, bosentan and valsartan treatment can dose-dependently downregulate the levels of CAR mRNA and protein which is required for viral entry into cells. Reduction of CAR significantly reduced viral load in CVB3-infected Human Umbilical Vein Endothelial Cells (HUVECs) (98, 99). What's more, House Dust Mite Specific Antibodies, which can bind to cardiac vascular endothelial cells to weaken their barrier function, have been detected in the serum of patients with myocarditis. Since barrier dysfunction may lead to local tissue inflammation, removal of this antibody may be effective in relieving myocarditis (100). Moreover, compared with healthy rats, rats with autoimmune myocarditis exhibited marked mobilization of Endothelial Progenitor Cells (EPCs) from the bone marrow to the periphery, and their ability to adhere to fibronectin, mature endothelial cells, and cultured cardiomyocytes was significantly reduced. Metastasis of splenocyte-derived EPCs partially attenuates myocardial injury induced by experimental myocarditis. Of these, the transformation of EPCs into resident mature endothelial cells appears to be the most important reason (101). Therefore, augmentation of damaged endothelial cells by cell therapy could also serve as a promising therapeutic approach.

Among endothelial cell-derived proteins with altered secretion or expression in myocarditis, depletion of functional selectin ligand expression reduces the migration of pathogenic CD8^+^ T cells to the heart, which in turn reduces cardiac inflammatory infiltration and cardiomyocyte damage in myocarditis. In addition, the expression of MHC class II in myocarditis endothelial cells with dilated cardiomyopathy is increased (102). Studies have shown that loss of MHC class II expression on endothelial cells protects mice from experimental autoimmune myocarditis (103). Cyclosporine and statins reduce endothelial MHC class II levels and may prevent diffuse endothelial dysfunction in myocarditis (98). What's more, the antisense ICAM-1 inhibitor alicaforsen has been studied in four Phase 2 studies in ulcerative colitis (UC), and analysis suggests that alicaforsen enema may provide an effective and potential lasting response (104). However, it still needs more evidence to confirm whether these drugs can affect the expression of ICAM-1 in endothelial cells in myocarditis and exert their therapeutic effect on myocarditis.

In conclusion, the factors that cause the activation of fibroblasts and endothelial cells or the active substances produced by fibroblasts or endothelial cells that affect the occurrence and development of myocarditis can be used as potential targets for clinical treatment of myocarditis.

## Conclusions

CFs and ECs can be infected by pathogens and release immunologically active substances to participate in the inflammatory response. In addition, CFs affect the fibrosis process and long-term prognosis of the heart through ECM changes. At the same time, through the interaction with the CMs, they may play a synergistic or antagonistic effect during the disease process.

However, there are still many problems. First of all, many of the interactions between cells are experiments conducted *in vitro*, such as the use of conditioned media or co-cultivation. It's difficult to perform *in vivo* experiments due to technical problems. However, the *in vitro* environment is quite different from the *in vivo* environment which has a more abundant neurohumoral regulation process.

In addition, a problem faced by cell experiments under *in vitro* conditions is that the conditioned medium may not be able to reproduce the observed effect. The composition and content of the conditioned medium will vary with the intervention environment, such as cell content, cell viability, cell intervention time, and the selection of cell lines, etc. That will make the results unstable.

Finally, the body is in a complex internal environment. The virus infection or the release of inflammatory factors both have beneficial and harmful aspects. This is closely related to the time and space relationship between cells. How to balance the pros and cons, and how to determine the correct intervention intensity, intervention time, and intervention methods is an important issue currently facing.

In general, a correct understanding of the function and mode of action of various cells, especially the non-inflammatory cells of the heart, such as CFs and ECs, will be more conducive to our understanding of diseases. And it contributes to the development of new myocarditis pathogenesis and corresponding treatment strategies.

## Author Contributions

DWW conceived this review, organized, and critically revised the manuscript. YX did major work of writing the manuscript. CC made contributions to editing the manuscript. ZW gave final approval of the version to be published. All authors were fully aware of and approved the submission of this manuscript.

## Funding

This work was supported by grants from the National Natural Science Foundation of China (81630010 and 81790624 to DWW, 82070395 to ZW), the Natural Science Foundation of Hubei Province (2020CFA016 to CC), and the Tongji Hospital Clinical Research Flagship Program (2019CR207 to DWW). No funding bodies had any role in the program design, preparation of the manuscript, or decision to publish.

## Conflict of Interest

The authors declare that the research was conducted in the absence of any commercial or financial relationships that could be construed as a potential conflict of interest.

## Publisher's Note

All claims expressed in this article are solely those of the authors and do not necessarily represent those of their affiliated organizations, or those of the publisher, the editors and the reviewers. Any product that may be evaluated in this article, or claim that may be made by its manufacturer, is not guaranteed or endorsed by the publisher.
